# New Insights into Phylogenetic Relationship of *Hydrocotyle* (Araliaceae) Based on Plastid Genomes

**DOI:** 10.3390/ijms242316629

**Published:** 2023-11-22

**Authors:** Rongrong Yan, Li Gu, Lu Qu, Xiaoyu Wang, Guoxiong Hu

**Affiliations:** 1 The Key Laboratory of Plant Resource Conservation and Germplasm Innovation in Mountainous Region, Ministry of Education, Guizhou University, Guiyang 550025, China; yanrrgzu@163.com (R.Y.); guligz@163.com (L.G.); gzuwangxiaoyu@163.com (X.W.); 2 College of Life Sciences, Guizhou University, Guiyang 550025, China; 3 Institute of Medicinal Plant Development Yunnan Branch, Chinese Academy of Medical Sciences, Jinghong 666100, China; ql199510@163.com

**Keywords:** *Hydrocotyle*, Hydrocotyloideae, phylogeny, comparative analysis, chloroplast genome, molecular markers

## Abstract

*Hydrocotyle*, belonging to the Hydrocotyloideae of Araliaceae, consists of 95 perennial and 35 annual species. Due to the lack of stable diagnostic morphological characteristics and high-resolution molecular markers, the phylogenetic relationships of *Hydrocotyle* need to be further investigated. In this study, we newly sequenced and assembled 13 whole plastid genomes of *Hydrocotyle* and performed comparative plastid genomic analyses with four previously published *Hydrocotyle* plastomes and phylogenomic analyses within Araliaceae. The plastid genomes of *Hydrocotyle* exhibited typical quadripartite structures with lengths from 152,659 bp to 153,669 bp, comprising a large single-copy (LSC) region (83,958–84,792 bp), a small single-copy (SSC) region (18,585–18,768 bp), and a pair of inverted repeats (IRs) (25,058–25,145 bp). Each plastome encoded 113 unique genes, containing 79 protein-coding genes, 30 tRNA genes, and four rRNA genes. Comparative analyses showed that the IR boundaries of *Hydrocotyle* plastomes were highly similar, and the coding and IR regions exhibited more conserved than non-coding and single-copy (SC) regions. A total of 2932 simple sequence repeats and 520 long sequence repeats were identified, with specificity in the number and distribution of repeat sequences. Six hypervariable regions were screened from the SC region, including four intergenic spacers (IGS) (*ycf3-trnS*, *trnS-rps4*, *petA-psbJ*, and *ndhF-rpl32*) and two coding genes (*rpl16* and *ycf1*). Three protein-coding genes (*atpE*, *rpl16*, and *ycf2*) were subjected to positive selection only in a few species, implying that most protein-coding genes were relatively conserved during the plastid evolutionary process. Plastid phylogenomic analyses supported the treatment of *Hydrocotyle* from Apiaceae to Araliaceae, and topologies with a high resolution indicated that plastome data can be further used in the comprehensive phylogenetic research of *Hydrocotyle*. The diagnostic characteristics currently used in *Hydrocotyle* may not accurately reflect the phylogenetic relationships of this genus, and new taxonomic characteristics may need to be evaluated and selected in combination with more comprehensive molecular phylogenetic results.

## 1. Introduction

Current molecular phylogenetic analyses indicate that *Hydrocotyle* belongs to the subfamily Hydrocotyloideae of Araliaceae [1,2,3,4]. This genus, comprising approximately 130 species, is widely distributed worldwide but primarily found in the Neotropics, Africa, Southeast Asia, and the warmer regions of China, with a few occurring in Europe [4,5,6]. The majority of the members of *Hydrocotyle* are perennial, but approximately 35 species endemic to Australia are annual herbs [4]. In China, all taxa, including 18 species, two subspecies, one variety, and two introduced species (*Hydrocotyle ranunculoides* and *H. verticillata*), that have been recorded are perennial [7,8]. *Hydrocotyle* is of economic importance and some species can be used for ornamental, medicinal, and edible purposes. For example, *H. verticillata* and *H. sibthorpioides* ‘Variegata’ have been widely used in wetland and aquatic landscape construction [9]. As a traditional Chinese herbal medicine, *H. sibthorpioides* contains flavonoids, glycerin, and triterpenes that can be used for the treatment of hepatitis and tumors and clearing away heat and toxic materials [10].

Historically, *Hydrocotyle* is a taxonomically difficult genus in which species differentiation relies heavily on the morphology of the fruit, inflorescence, and leaf [11,12,13]. However, many morphologically similar taxa show continuous variation, which makes accurate identification very difficult between or within species. For example, *Hydrocotyle sibthorpioides* var. *sibthorpioides* is morphologically similar to *H. sibthorpioides* var. *batrachium*. The main difference is that the leaves of the former are shallowly 5–7 lobed or nearly entire, while the leaves of the latter are deeply 3–5 lobed [7]. In our field investigations, however, there is continuous variation in the degree of leaf division, whereby 3–7 lobed leaves can be seen in the same population. In addition, *H. hamelinensis* and *H. puberula* were previously recognized as two putatively distinct species on the basis of morphology in Australia but were finally demonstrated to be conspecific with *H. tetragonocarpa* and *H. scutellifera*, respectively [4]. Recently, simple DNA markers, such as ITS, *matK*, *rpl16*, *trnD-trnT*, and *psbA-trnH*, have been applied to solve the phylogenetic relationships of *Hydrocotyle* [2,4,14,15]. Although these short DNA fragments can recognize the main clades within *Hydrocotyle*, topologies among closely related species are still polytomous or have low phylogenetic resolution.

Compared with the complex nuclear genome and unstable mitochondrial genome, the plastid genome shows great advantages, such as uniparental inheritance, a stable genetic structure, and a relatively small size for genetic and evolutionary analyses [16,17,18]. At present, complete plastid genomes are widely being applied to solve phylogenetic relationships at different taxonomical levels within angiosperms [19,20,21,22] and have significantly improved the phylogenetic resolution of Araliaceae compared with Sanger sequencing data [23,24,25,26]. To date, four complete plastid genomes of *Hydrocotyle* have been reported, including *Hydrocotyle nepalensis*, *H. pseudoconferta*, *H. sibthorpioides*, and *H. verticillata*. Previous studies have placed a greater focus on the plastome size and gene content for a single species of *Hydrocotyle* [27,28], but plastid comparative genomes and phylogenomic analyses at the generic level are still lacking. Given the importance of comprehensive genomics to understand plastome evolution and phylogenetic relationships in *Hydrocotyle*, even Araliaceae, it is necessary to analyze more plastid genomes of *Hydrocotyle*.

In this study, we newly sequenced and assembled 13 plastid genomes of *Hydrocotyle*. Comparative genomic analyses were conducted for 17 *Hydrocotyle* plastomes and phylogenomic analyses were performed within Araliaceae. Our aims were to (1) analyze the structural features and sequence variations of *Hydrocotyle* plastomes; (2) detect repeat sequences and highly variable regions as potential molecular markers; and (3) infer the phylogenetic relationships among *Hydrocotyle* species based on the whole plastid data.

## 2. Results

### 2.1. Plastome Structure and Features

**Figure 1:**
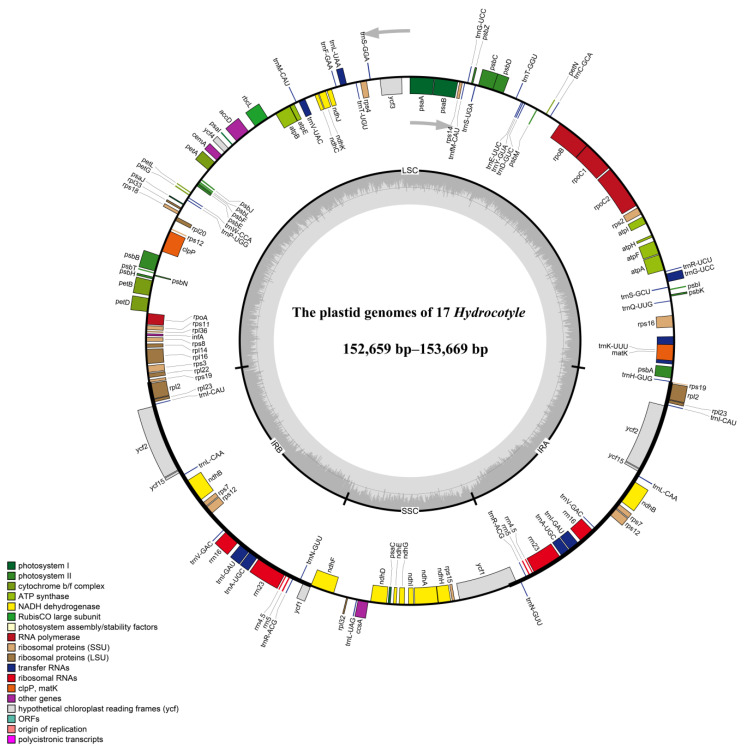
Gene map of the plastid genomes of *Hydrocotyle*. Genes with different functions are coded by color. Genes shown inside the inner circle are transcribed clockwise, and those outside are transcribed counterclockwise. The dark gray color in the inner circle indicates GC content, while the light gray color corresponds to AT content. LSC, large single copy; SSC, small single copy; IR, inverted repeat.

The complete plastid genomes of the 17 *Hydrocotyle* taxa ranged from 152,659 bp (*Hydrocotyle sibthorpioides* ‘Variegata’) to 153,669 bp (*H. ranunculoides*) in size (Table 1). These plastomes exhibited a typical quadripartite structure, comprising a large single-copy (LSC) region (83,958–84,792 bp), a small single-copy (SSC) region (18,585–18,768 bp), and a pair of inverted repeats (IRs) (25,058–25,145 bp) (Table 1 and Figure 1). The total GC content showed slight variation (37.50–37.67%), with an uneven distribution in the plastid genomes, of which the IR region was highest (43.08–43.30%), followed by the LSC region (35.52–35.70%) and the SSC region (30.95–31.22%).

The gene content was identical among these *Hydrocotyle* plastomes. A total of 134 genes were annotated, containing 85 protein-coding genes (PCGs), 37 tRNA genes, eight rRNA genes, and four pseudogenes (Table 2). Among these, seven tRNA genes (*trnA-UGC*, *trnI-CAU*, *trnI-GAU*, *trnL-CAA*, *trnN-GUU*, *trnR-ACG*, and *trnV-GAC*), six protein-coding genes (*ndhB*, *rpl2*, *rpl23*, *rps7*, *rps12*, and *ycf2*), and four rRNA genes (*rrn4.5*, *rrn5*, *rrn16*, and *rrn23*) were completely duplicated in the IR region. In all *Hydrocotyle* plastomes, the incompletely duplicated copies of *rps19* in the IRa region and *ycf1* in the IRb region were identified as pseudogenes. Since the stop codon appeared prematurely, the gene *ycf15* in two IR regions was annotated as a pseudogene. There were 17 genes with introns, of which three genes (*clpP*, *rps12*, and *ycf3*) contained two introns and the other 14 genes possessed a single intron.

### 2.2. Comparative Genomics and Hypervariable Regions

The gene composition for each IR boundary exhibited high similarity, with slight variations in gene arrangement patterns (Supplementary Figure S1). The LSC/IRb junction was located in the coding region of gene *rps19*, with an incomplete copy (62 bp) in the IRa region, forming a pseudogene (Ψ*rps19*). The SSC/IRb junction was crossed by the partial *ycf1* gene, which ranged from 764 bp to 774 bp in the IRb region, and the remaining part of the *ycf1* gene expanded into the SSC regions from 4 bp to 76 bp, overlapping with the *ndhF* gene. Meanwhile, the gene *ndhF* spanned the SSC/IRb junction, and the length of this gene extending into the IRb region ranged from 28 bp to 70 bp. The SSC/IRa junction was located in the complete *ycf1* gene, with 4679 bp to 4759 bp in the SSC region and 764 bp to 774 bp in the IRa region. At the LSC/IRa junction, the *rpl2* and *trnH* genes were entirely distributed within the LSC and IRa regions near the junction.

**Figure 2:**
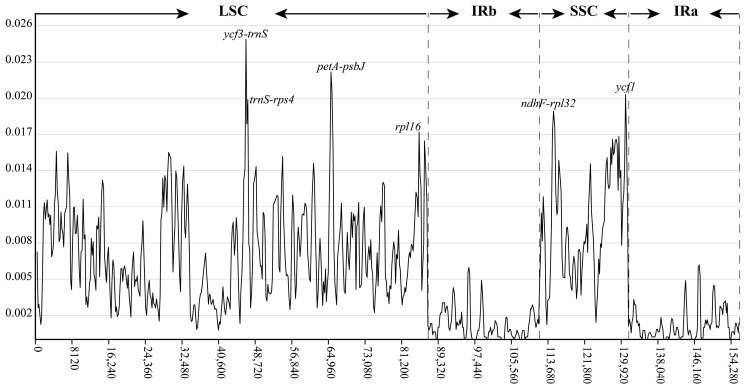
Sliding window analysis of the 17 *Hydrocotyle* plastomes. The X-axis represents the position of the midpoint of a window, and the Y-axis indicates the nucleotide diversity (Pi) of each window.

No gene rearrangements were detected in the plastid genomes of *Hydrocotyle*, demonstrating that their genome structure was highly conserved (Supplementary Figure S2). The mVISTA analyses showed that the single-copy (SC) and non-coding regions were more divergent than the IR and coding regions (Supplementary Figure S3). To further identify hypervariable regions, we calculated the nucleotide variability (Pi) using sliding window analysis. The Pi values ranged from 0 to 0.02488 in the whole plastid genomes, with an average of 0.005459 (Figure 2). A total of six hypervariable regions were identified (Pi > 0.01700), including four intergenic spacers (IGS) (*ycf3-trnS*, *trnS-rps4*, *petA-psbJ*, and *ndhF-rpl32*) and two coding genes (*rpl16* and *ycf1*), all of which were from the SC region.

### 2.3. Repeat Sequence Characteristics

**Figure 3:**
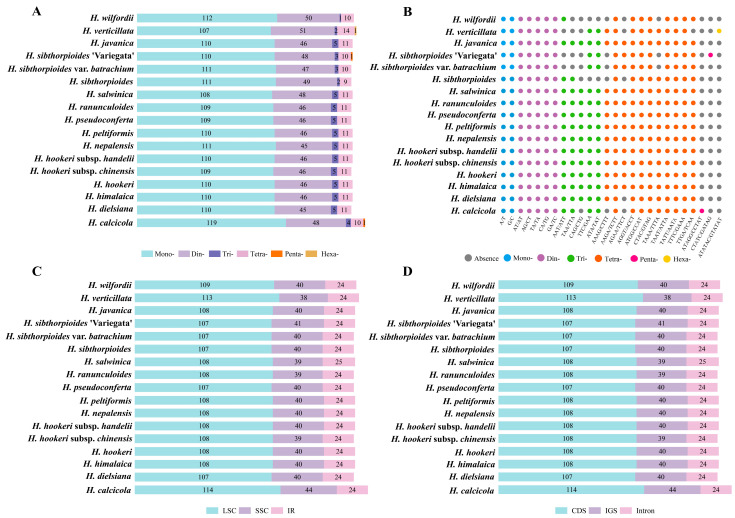
Analyses of simple sequence repeats (SSRs) among 17 *Hydrocotyle* plastomes. (**A**), Numbers of different types of SSRs. (**B**), Types of shared SSRs. (**C**), Distributions of SSRs in the LSC, SSC, and IR regions. (**D**), Distributions of SSRs in the CDS, IGS, and intron.

Simple sequence repeats (SSRs) were detected among the 17 *Hydrocotyle* taxa, with numbers ranging from 171 to 182 (Supplementary Table S1). Mononucleotide repeats accounted for the most part (1876, 63.98%), followed by the dinucleotide (799, 27.25%), the tetranucleotide (184, 6.28%), and the trinucleotide repeats (70, 2.39%). Pentanucleotide repeats were only detected in *Hydrocotyle calcicola* and *H. sibthorpioides* ‘Variegata’. Hexanucleotide repeats were present only in *H. verticillata* (Figure 3A). Two types of mononucleotide repeats and five types of dinucleotide repeats were detected in all species (Figure 3B). Of these, the A/T base was the most abundant, and the dinucleotide repeats were mainly concentrated in the AT/AT base (Supplementary Table S1). The majority of SSRs were located in the LSC region (1843, 62.86%) compared with the SSC (680, 23.19%) and IR (409, 13.95%) regions (Figure 3C). Furthermore, SSRs were distributed mostly in the coding sequence (CDS) (1230, 41.95%) and IGS (1406, 47.95) regions, with a few in the introns (296, 10.10%) (Figure 3D).

**Figure 4:**
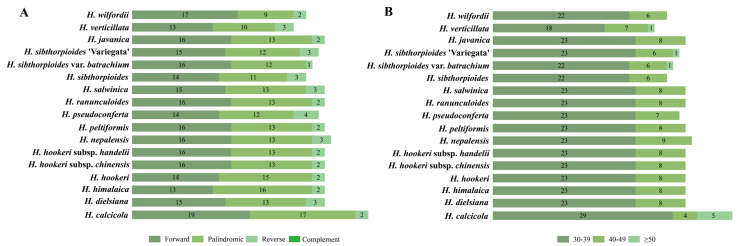

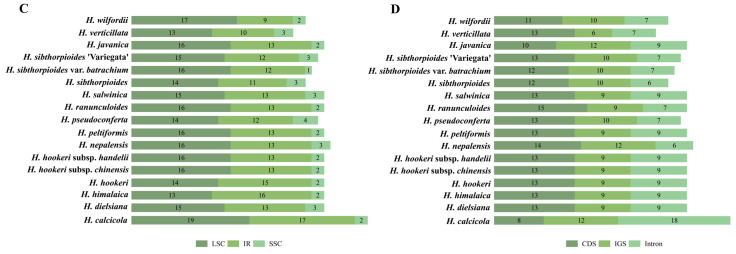
Analyses of long sequence repeats (LSRs) among 17 *Hydrocotyle* plastomes. (**A**) Numbers of different types of LSRs. (**B**) Numbers of different lengths of LSRs. (**C**) Distributions of LSRs in the LSC, SSC, and IR regions. (**D**) Distributions of LSRs in the CDS, IGS, and intron.

Three types of long sequence repeats (LSRs) were recorded, including 251 forward, 252 palindromic, and 17 reverse repeats (Supplementary Table S2). The numbers of LSRs ranged from 26 to 38, of which forward and palindromic repeats were detected in all taxa, but reverse repeats were not detected in *Hydrocotyle hookeri*, *H. hookeri* subsp. *chinensis*, *H. hookeri* subsp. *handelii*, *H. javanica*, *H. peltiformis*, and *H. ranunculoides* (Figure 4A). In terms of length, most repeat sequences consisted of 30 bp to 39 bp, followed by 40 bp to 49 bp, with very few sequences greater than 50 bp (Supplementary Table S2 and Figure 4B). These repeat sequences were uneven across different regions, with 261 in the LSC regions, 218 in the IR regions, and 41 in the SSC regions (Figure 4C). Most of the LSRs were scattered in the IGS (164, 31.54%) and CDS (212, 40.77%) regions, with a few in the introns (144, 27.69%) (Figure 4D).

### 2.4. Codon Usage and Selective Pressure

Similarity in amino acid frequency and codon usage was observed among the 17 *Hydrocotyle* plastomes (Supplementary Table S3). The number of codons ranged from 25,960 (*Hydrocotyle sibthorpioides*) to 26,178 (*H. ranunculoides* and *H. salwinica*), encoding 20 amino acids. Of these, leucine (Leu) was the most abundant amino acid (10.6% in each taxon), and cysteine (Cys) was the least common (1.1–1.2%). A total of 64 types of codons were detected, of which most amino acids were encoded by more than one codon, except for methionine (Met) and tryptophan (Trp), which only had a single codon. The highest and lowest relative synonymous codon usage (RSCU) values were from UUA (1.85–1.89) and AGC (0.37–0.39), encoding Leu and serine (Ser), respectively. Moreover, 30 codons showed usage bias with RSCU greater than 1, all of which ended with A or U, except for UUG. In contrast, most codons ended with G or C with RSCU values of less than 1, indicating codon usage with less preference. Among the three stop codons (UAA, UAG, and UGA), codon usage showed a bias toward UAA (1.34–1.45).

Non-synonymous (Ka) substitutions, synonymous (Ks) substitutions, and their ratio (Ka/Ks) were calculated based on 79 protein-coding genes (Supplementary Table S4). For the majority of protein-coding genes, the Ka/Ks values were less than 1, meaning that most genes were subjected to purifying selection. The Ka/Ks values of three genes (*atpE*, *rpl16*, and *ycf2*) were greater than 1, implying potentially positive selection. Among these, the *rpl16* gene was found with positive selection in *Hydrocotyle dielsiana* (Ka/Ks = 1.0081), *H. himalaica* (Ka/Ks = 1.0081), *H. hookeri* (Ka/Ks = 1.0081), *H. hookeri* subsp. *chinensis* (Ka/Ks = 1.0081), *H. hookeri* subsp. *handelii* (Ka/Ks = 1.0081), *H. javanica* (Ka/Ks = 1.0081), *H. nepalensis* (Ka/Ks = 1.0081), *H. peltiformis* (Ka/Ks = 1.3440), *H. pseudoconferta* (Ka/Ks = 1.0081), and *H. salwinica* (Ka/Ks = 1.0081). The genes *atpE* (Ka/Ks = 2.3884) and *ycf2* (Ka/Ks = 1.0646) with positive selection were detected only in *H. ranunculoides*.

### 2.5. Phylogenetic Relationships

**Figure 5:**
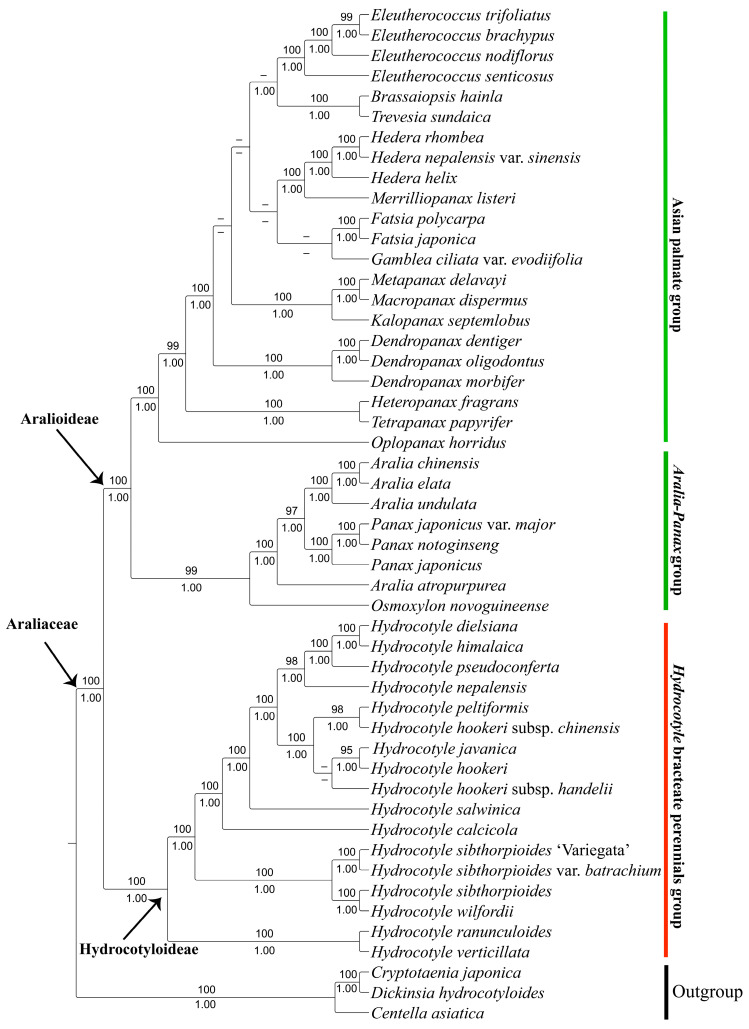
Phylogenetic tree of *Hydrocotyle* and related taxa inferred from the whole plastid genomes using maximum likelihood (ML) and Bayesian inference (BI) methods. The numbers above and below the branches are the ML bootstrap values (BS) and BI posterior probabilities (PP), respectively. BS < 50 and PP < 0.9 are represented by “–”.

**Figure 6:**
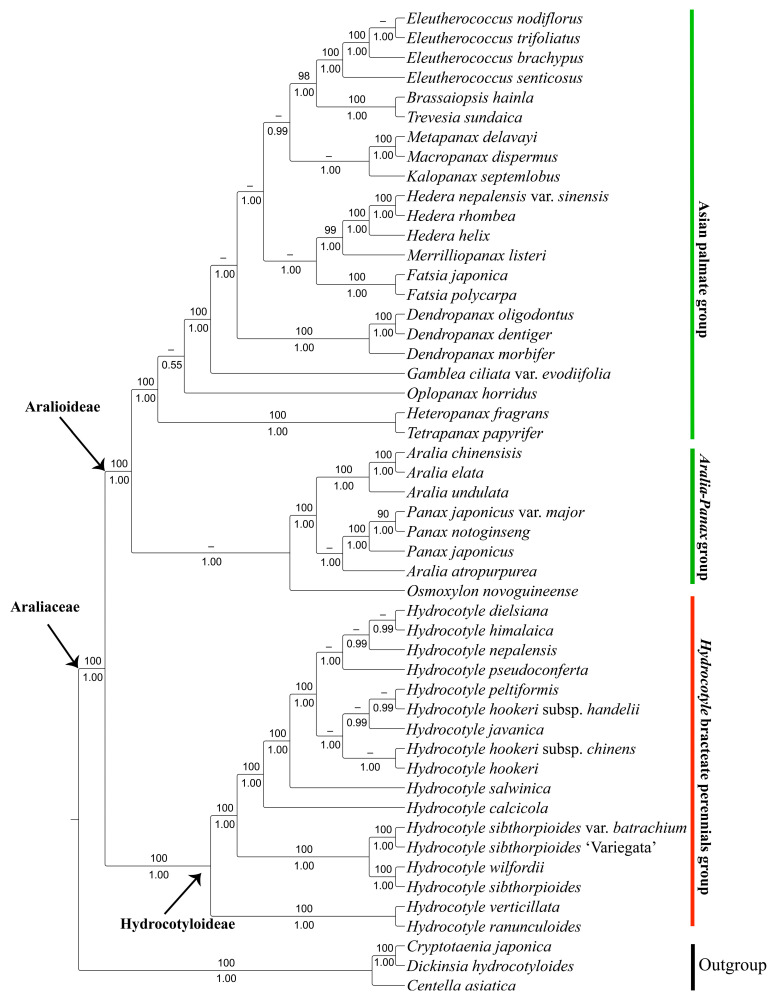
Phylogenetic tree of *Hydrocotyle* and related taxa inferred from protein-coding genes using maximum likelihood (ML) and Bayesian inference (BI) methods. The numbers above and below the branches are the ML bootstrap values (BS) and BI posterior probabilities (PP), respectively. BS < 50 and PP < 0.9 are represented by “–”.

To further infer the evolutionary relationships of *Hydrocotyle*, phylogenetic trees were constructed based on the whole plastid genomes and shared protein-coding genes using the maximum likelihood (ML) and Bayesian inference (BI) methods. The alignment of plastomes yielded a matrix of 150,488 bp, comprising 126,586 constant sites and 11,861 parsimony-informative sites. The aligned protein-coding genes comprised 56,233 bp, of which 49,065 bp were constant sites and 3332 bp were parsimony-informative sites. As the topologies inferred from ML and BI analyses were almost identical, only the phylogram of the ML method was presented with bootstrap support (BS) and posterior probability (PP) values added (Figure 5 and Figure 6). Overall, the supported values based on the whole plastid sequences for each node were much higher than those inferred from protein-coding genes.

Phylogenomic analyses based on the completed plastid genome and protein-coding genes both supported the monophyly of the two subfamilies within the family Araliaceae (BS = 100, PP = 1.00). In subfamily Aralioideae, the sister relationship between the Asian palmate group and the *Aralia-panax* group was strongly supported (BS = 100, PP = 1.00). In subfamily Hydrocotyloideae, *Hydrocotyle* was a well-supported monophyletic taxon (BS = 100, PP = 1.00), in which two subclades were recognized. One comprised two species native to the Neotropics (*Hydrocotyle verticillata* and *H. ranunculoides*), sister to another subclade consisting of taxa from China. Within the Chinese subclade, *H. sibthorpioides*, including its variety (*H. sibthorpioides* var. *batrachium*) and cultivar (*H. sibthorpioides* ‘Variegata’), formed a group together with *H. wilfordii*. Another group included 11 taxa with *H. calcicola* sister to the remaining 10 taxa.

## 3. Discussion

### 3.1. Structural Features and Plastome Evolution

As with most angiosperms [29,30,31,32], the plastid genomes of *Hydrocotyle* were relatively conserved, with a similar quadripartite structure, gene order, gene content, and GC content (Figure 1 and Table 1). Generally, the expansion and contraction of inverted repeat (IR) regions are common and may lead to size variation, gene loss, and pseudogenization, which play a crucial role in genome evolution [18]. In *Hydrocotyle*, the plastome size ranged from 152,659 bp to 153,669 bp, with no significant variations observed. The IR boundaries showed high similarity among all species (Supplementary Figure S1), indicating that their plastid genomic structure was highly conserved. Compared with other genera, such as *Dendropanax* [26], *Eleutherococcus* [33], and *Panax* [34] of Araliaceae, the small single-copy (SSC) region of the *Hydrocotyle* plastomes exhibited a slight expansion due to the *ndhF* gene extending into the IRb region. The pseudogenization of both shorter copies (*rps19* and *ycf1*) was caused by incomplete duplication, which has also occurred in most Araliaceae plants [23,25,27]. Furthermore, the *ycf15* gene containing premature stop codons was annotated as a pseudogene across the 17 *Hydrocotyle* plastomes. In some Araliaceae plastomes, however, the *ycf15* was intact [23,25,33] or completely lost [26,35], demonstrating that a variation in gene content has occurred during the process of evolution. Although pseudogenes have lost their ability to encode proteins, they are still transcribed and have great significance for regulatory processes [36,37]. The *ycf15* gene, a hypothetical open reading frame, is distributed in the IR regions among *Hydrocotyle* plastomes, but its function remains unknown and needs to be further investigated.

Codon usage bias is closely related to gene expression and plants’ adaptation to their environments [38]. Genes with high expression guarantee the efficient recycling of the ribosomes through the use of well-adapted codons to improve cellular fitness [39]. In *Hydrocotyle* plastomes, the relative synonymous codon usage (RSCU) analyses indicated that most codons showed a strong preference toward A/U-ending synonymous codons (RSCU > 1) (Supplementary Table S3). This is a common phenomenon in the plastid genomes of most land plants and may be correlated with the GC content of codons [40,41,42,43]. Overall, the codon usage pattern presented high similarity among *Hydrocotyle* plastomes, implying that gene expression is relatively conserved at the plastid level. Moreover, non-synonymous (Ka) substitutions, synonymous (Ks) substitutions, and their ratios (Ka/Ks) are widely used to evaluate selective pressure on protein-coding genes [44]. Previous studies have indicated that most genes are subjected to purifying selection to retain conserved functions throughout plastid evolution [45,46,47]. Similar to previous studies [48,49], most protein-coding genes (76 out of 79 genes) had Ka/Ks values less than 1, of which the Ka/Ks values of photosynthesis-related genes were significantly lower than those of other genes (Supplementary Table S4). In the present study, three genes were likely to undergo positive selection (Ka/Ks > 1), including the subunit of ATP synthase gene *atpE*, the large subunit of ribosome gene *rpl16*, and the unknown function gene *ycf2*. The *atpE* gene encoding ε subunits of ATP synthase plays an important role in plant photosynthesis, and positive selection on the *atpE* gene has also been reported in Cucurbitaceae [50], Liliaceae [51], and Zingiberaceae [52]. The *rpl16* gene encodes the ribosomal protein L16, which has been proven to be essential in plastid ribosome development [53]. It is worth mentioning that this highly conserved gene exhibiting positive selection needs to be confirmed for future research [54]. As the largest known plastid gene, *ycf2* is essential for cell viability but of unknown function [55], presenting positive selection signals in most land plants [22,48,56,57,58]. Given that genes with positive selection may be undergoing adaptive evolution [59], it is necessary to further explore the selective pressure among *Hydrocotyle* species based on more extensive sampling with phylogenetic clades.

### 3.2. Sequence Polymorphisms and Hypervariable Regions

The implementation of simple sequence repeat (SSR) molecular markers is characterized by low costs, high polymorphism, reproducibility, and transferability across species [60], and they are widely applied in population genetics [61]. In most land plants, the distribution of SSRs in plastid genomes is uneven, with most of them found in the coding and non-coding regions, with a few in introns [62,63,64], which was confirmed in the current study (Figure 3). The lower number of SSRs in the IR region compared with the single-copy (SC) region may be related to the slower evolutionary rate in the IR region [65]. Consistent with previous studies [66,67], mononucleotide repeats were the most abundant type and primarily dominated by the A/T base. Furthermore, long sequence repeats (LSRs) have great significance for genome recombination and rearrangement [68]. We detected three types of LSRs among *Hydrocotyle* species, including forward, palindromic, and reverse repeats, of which forward and palindromic repeats were the most common (Figure 4), in line with previous studies [22,46,48,63]. The number and distribution of LSRs presented differences among different taxa, which may be one of the reasons for the diversity of plastid genomes at the generic level [69].

Hypervariable regions screened from the plastid genome can be used as potential DNA barcodes or genetic markers for species identification, biogeographic inference, and phylogenetic analyses [70]. Based on a few universal DNA fragments, however, the phylogenetic topologies of *Hydrocotyle* among closely related species were polytomous or had a low resolution [2,4,14,15]. Due to the taxonomically challenging and high medicinal and ornamental value, lineage-specific molecular markers are needed to avoid misidentification for *Hydrocotyle* species. Previous plastome studies have mainly focused on the size and gene content of a single species of *Hydrocotyle* [27,28], lacking detailed comparative genomics. In this study, six hypervariable regions were identified among 17 *Hydrocotyle* plastomes, including four intergenic spacers (IGS) (*ycf3-trnS*, *trnS-rps4*, *petA-psbJ*, and *ndhF-rpl32*) and two coding genes (*rpl16* and *ycf1*), all of which were from the SC region. Of these, *ycf3-trnS* showed the highest Pi value (0.02488) and was also recognized as a candidate barcode in *Dendropanax* of Araliaceae [26]. These hypervariable regions provide new insights into the development of DNA barcodes for *Hydrocotyle*, but further research is needed to determine which of these regions are suitable for species delimitation and identification.

### 3.3. Phylogenomic Analyses

For a long time, molecular phylogenetic analyses inferred from Sanger sequencing data failed to clearly elucidate the phylogenetic relationships of *Hydrocotyle* [2,14,15]. In the current study, the phylogenetic trees of *Hydrocotyle* reconstructed based on whole plastid genomes and protein-coding genes showed a high phylogenetic resolution, in contrast with previous studies using a few molecular markers (Figure 5 and Figure 6). All topologies strongly supported the monophyly of the subfamily Hydrocotyloideae and Aralioideae (BS = 100, PP = 1.00). Historically, Drude [71] placed Hydrocotyloideae in Apiaceae based on the superficial characteristics of the fruit. On the basis of molecular phylogenetic results with more detailed morphological studies, the traditionally defined Hydrocotyloideae was demonstrated to be polyphyletic and some genera (e.g., *Hydrocotyle*, *Trachymene*, *Neosciadium*, and *Uldinia*) have been transferred from Apiaceae to Araliaceae [1,2,72,73]. In this study, plastid phylogenomic analyses also supported this treatment.

Phylogenomic analyses indicated that the Chinese *Hydrocotyle* formed two independent lineages (Figure 5 and Figure 6). The result is different from the taxonomic treatment based on morphological evidence. Within *Hydrocotyle,* the length of the petiole and pedicel/peduncle as well as the size of the leaf blade are regarded as the main diagnostic characteristics [7]. Based on the smaller size of the leaf blade (0.5–1.5 × 0.8–2 cm), *Hydrocotyle pseudoconferta*, *H. sibthorpioides*, and *H. calcicola* are considered to be closely related taxa. However, in our phylogenomic analyses, the three species did not group, with *H. pseudoconferta* sister to *H. himalaica* + *H. dielsiana*, *H. sibthorpioides* sister to *H. wilfordii*, and *H. calcicola* forming an isolated clade. Similarly, on the basis of the synapomorphies of the elongated pedicel (2.5–8 mm) and undensely capitate umbel, *H. dielsiana*, *H. himalaica*, and *H. hookeri* should have gathered in a lineage, but phylogenomic analyses showed that the three species were scattered in two different branches. Traditionally, taxonomic treatment mainly relies on morphological characteristics. However, the selection of taxonomic characteristics is subjective, and different researchers may choose different characteristics, leading to different taxonomic results for the same taxa. When inferring phylogenetic relationships, molecular systematic evidence is often more convincing than morphological evidence. For example, the genera *Callicarpa*, *Clerodendrum*, and *Vitex* were placed in the Verbenaceae because they lack four dry nutlets, but molecular systematic studies have confirmed that these genera with cymose inflorescences should be transferred to the Lamiaceae [21,74]. Therefore, the main diagnostic characteristics applied in the taxonomic treatment of *Hydrocotyle* may need to be reevaluated, and new diagnostic characteristics should be screened in combination with more comprehensive molecular phylogenetic results.

## 4. Materials and Methods

### 4.1. Plant Material, DNA Extraction, and Sequencing

Plant samples were collected from the field and the voucher specimens were deposited in the herbarium of the Natural Museum of Guizhou University (GACP). Details of the sampling information and vouchers are shown in Table 3. Total genomic DNA was extracted from the fresh or silica-gel-dried leaves using the modified cetyltrimethylammonium bromide (CTAB) method [75]. DNA integrity and concentration were monitored with agarose gel electrophoresis and a Qubit Fluorometer, respectively. Total DNA was fragmented randomly to construct shotgun libraries, and then qualified libraries were selected for paired-end (150 bp) sequencing by the Illumina Hiseq 2500 platform at Wuhan BGI Technology Service Co., Ltd. (Wuhan, China). The low-quality reads of raw data were filtered using SOAPnuke v.2.0 [76], generating 3–5 GB of clean data for each taxon.

### 4.2. Plastome Assembly and Annotation

GetOrganelle v.1.6.2 [77] was applied to assemble plastomes based on clean data with default parameters. The final assembly results were visualized and checked with Bandage v0.8.1 [78]. Plastid Genome Annotator (PGA) [79] was used to annotate the complete plastid genome with *Hydrocotyle sibthorpioides* (KT589392) as the reference, followed by the validation of the annotation using CPGAVAS2 [80]. Gene start and stop codons were checked and manually inspected in the software Geneious v.10.0.5 [81], and the tRNA genes were further verified using tRNAscan-SE v1.21 [82]. Finally, the plastid genome maps of *Hydrocotyle* were drawn with OrganellarGenomeDRAW (OGDRAW) https://chlorobox.mpimp-golm.mpg.de/OGDraw.html (accessed on 1 May 2023) [83]. 

### 4.3. Comparative Genomic Analyses

In total, 17 *Hydrocotyle* plastomes were included to perform comparative analyses, of which four plastids’ data (GenBank accession no.: MT561038, OK585058, KT589392, and HM596070) were downloaded from the GenBank database. The annotations of plastomes from GenBank were double-checked according to the above details and manually adjusted when necessary. Gene order and structure rearrangements were detected using Mauve v2.4.0 [84]. Sequence divergence was visualized based on the Shuffle-LAGAN model using mVISTA http://genome.lbl.gov/vista/mvista/submit.shtml (accessed on 10 May 2023) [85]. DnaSPv.6.12.03 [86] was used to calculate nucleotide diversity (Pi), setting the step size and window length to 200 bp and 600 bp, respectively. The expansion and contraction of inverted repeat (IR) regions were detected by the online program IRscope https://irscope.shinyapps.io/irapp/ (accessed on 10 May 2023) [87].

### 4.4. Repeat Sequence Analyses

Simple sequence repeats (SSRs) were detected using MISA https://webblast.ipk-gatersleben.de/misa/ (accessed on 15 May 2023) [88] by setting the parameters to eight, four, four, three, three, and three for mono-, di-, tri-, tetra-, penta-, and hexa-nucleotides, respectively. Long sequence repeats (LSRs), including forward (F), reverse (R), palindrome (P), and complement (C) repeats, were identified using REPuter https://bibiserv.cebitec.uni-bielefeld.de/reputer (accessed on 15 May 2023) [89], with a minimum repeat size of 30 bp and a Hamming distance of 3.

### 4.5. Codon Usage Bias and Selective Pressure Analyses

Amino acid frequency and relative synonymous codon usage (RSCU) were calculated using Codon W v.1.4.4 https://sourceforge.net/projects/codonw/ (accessed on 15 May 2023). The RSCU value was greater than 1, equal to 1, and less than 1, indicating preferred, no bias, and less preferred in codon usage, respectively. To assess selective pressure, 79 single-copy protein-coding genes were extracted using Geneious v.10.0.5 [81] after excluding duplicate genes and pseudogenes, and then aligned by MAFFT v.7.3888 [90]. Non-synonymous (Ka) and synonymous (Ks) substitutions were calculated using KaKs_Calculator 2.0 [91] under the Yang–Nielsen (YN) model [92], with *Hydrocotyle sibthorpioides* (KT589392) as the reference. The Ka/Ks value was greater than 1, equal to 1, and less than 1, demonstrating positive selection, neutral selection, and purifying selection, respectively.

### 4.6. Phylogenetic Analyses

Phylogenetic analyses including 50 taxa were performed based on the whole plastid genomes and protein-coding genes, of which 47 species from Araliaceae served as the ingroups and the other three species from Apiaceae as the outgroups (Supplementary Table S5). Sequences were aligned using MAFFT v.7.3888 [90] with the auto strategy and then manually adjusted by BioEdit v.7.0.9 [93]. Phylogenetic trees were constructed based on the maximum likelihood (ML) and Bayesian inference (BI) methods in CIPRES Science Gateway https://www.phylo.org/ (accessed on 28 May 2023). ModelFinder [94] was used to select the best-fit model according to the Akaike Information Criterion (AIC) [95]. The ML analyses were performed under the GTRGAMMA model with 1000 bootstrap replicates. The GTR + F + I + G4 model was selected for BI analyses with four parallel Markov chain Monte Carlo (MCMC) chains for 2,000,000 generations and sampling for every 1000 generations. After removing the first 25% of trees as burn-in, the remaining trees were used to generate the consensus tree. Finally, the phylogenetic results were visualized and edited in Figtree v.1.4.4 http://tree.bio.ed.ac.uk/software/figtree/ (accessed on 5 June 2023).

## 5. Conclusions

In this study, we newly sequenced and assembled 13 whole plastid genomes of *Hydrocotyle* and performed comparative plastid genome analyses with four previously published data and phylogenomic analyses within Araliaceae. Comparative analyses revealed that the *Hydrocotyle* plastomes were relatively conserved, with a similar quadripartite structure, gene order, gene content, and GC content. The boundaries of inverted repeats (IRs) and single-copy (SC) regions showed high similarity among all *Hydrocotyle* plastomes. Six hypervariable regions and 2932 simple sequence repeats were identified as potential molecular markers. Three genes (*atpE*, *rpl16*, and *ycf2*) were observed under positive selection only in a few taxa, implying that most protein-coding genes were relatively conserved and subjected to purifying selection during the plastid evolutionary process. Phylogenomic analyses supported transferring the genus *Hydrocotyle* from Apiaceae to Araliaceae, and the whole plastid genome was demonstrated to be effective in improving the phylogenetic resolution of *Hydrocotyle* in contrast with short DNA fragments. The diagnostic characteristics currently used in *Hydrocotyle* may not accurately reflect the phylogenetic relationships of this genus, and new diagnostic characteristics need to be screened based on more comprehensive molecular phylogenetic results.

## Figures and Tables

**Table 1:** Plastid genome characteristics of the 17 *Hydrocotyle* taxa.

Taxon	Length (bp)				GC Content (%)				Genes			
	Total	LSC	SSC	IR	Total	LSC	SSC	IR	PCGs	tRNA	rRNA	Pseudogene
*H. calcicola*	153,305	84,291	18,724	25,145	37.60	35.70	30.95	43.25	85	37	8	4
*H. dielsiana*	153,297	84,412	18,767	25,059	37.64	35.74	31.06	43.29	85	37	8	4
*H. himalaica*	153,299	84,413	18,768	25,059	37.64	35.74	31.05	43.29	85	37	8	4
*H. hookeri*	153,294	84,372	18,768	25,077	37.64	35.76	31.07	43.08	85	37	8	4
*H. hookeri* subsp. *chinensis*	153,289	84,388	18,747	25,077	37.64	35.76	31.08	43.27	85	37	8	4
*H. hookeri* subsp. *handelii*	153,323	84,403	18,766	25,077	37.64	35.75	31.07	43.27	85	37	8	4
*H. javanica*	153,324	84,403	18,767	25,077	37.63	35.75	31.07	43.27	85	37	8	4
*H. nepalensis* *	153,353	84,432	18,767	25,077	37.63	35.74	31.06	43.27	85	37	8	4
*H. peltiformis*	153,311	84,390	18,767	25,077	37.64	35.75	31.07	43.27	85	37	8	4
*H. pseudoconferta* *	153,302	84,417	18,767	25,059	37.64	35.74	31.07	43.29	85	37	8	4
*H. ranunculoides*	153,669	84,792	18,729	25,074	37.67	35.75	31.22	43.28	85	37	8	4
*H. salwinica*	153,294	84,372	18,768	25,077	37.65	35.76	31.07	43.27	85	37	8	4
*H. sibthorpioides* *	152,880	84,064	18,690	25,063	37.51	35.55	30.95	43.25	85	37	8	4
*H. sibthorpioides* var. *batrachium*	152,740	84,030	18,594	25,058	37.62	35.69	31.02	43.29	85	37	8	4
*H. sibthorpioides* ‘Variegata’	152,659	83,958	18,585	25,058	37.60	35.56	31.04	43.30	85	37	8	4
H. verticillata*	153,207	84,352	18,737	25,059	37.59	35.66	31.08	43.29	85	37	8	4
*H. wilfordii*	152,950	84,134	18,690	25,063	37.50	35.52	30.95	43.25	85	37	8	4

Note: The sequence from GenBank is indicated by *.

**Table 2:** Gene content and functional classification of the *Hydrocotyle* plastomes.

Category	Gene Group	Gene Name
Photosynthesis	Subunits of photosystem I	*psaA*, *psaB*, *psaC*, *psaI*, *psaJ*
	Subunits of photosystem II	*psbA*, *psbB*, *psbC*, *psbD*, *psbE*, *psbF*, *psbH*, *psbI*, *psbJ*, *psbK*, *psbL*, *psbM*, *psbN*, *psbT*, *psbZ*
	Subunits of NADH dehydrogenase	*ndhA* ^a^, *ndhB* ^a^ (2), *ndhC*, *ndhD*, *ndhE*, *ndhF*, *ndhG*, *ndhH*, *ndhI*, *ndhJ*, *ndhK*
	Subunits of cytochrome b/f complex	*petA*, *petB* ^a^, *petD* ^a^, *petG*, *petL*, *petN*
	Subunits of ATP synthase	*atpA*, *atpB*, *atpE*, *atpF* ^a^, *atpH*, *atpI*
	Large subunit of rubisco	*rbcL*
Self-replication	Proteins of large ribosomal subunit	*rpl14*, *rpl16* ^a^, *rpl2* ^a^ (2), *rpl20*, *rpl22*, *rpl23*(2), *rpl32*, *rpl33*, *rpl36*
	Proteins of small ribosomal subunit	Ψ*rps19*, *rps11*, *rps12* ^b^ (2), *rps14*, *rps15*, *rps16* ^a^, *rps18*, *rps19*, *rps2*, *rps3*, *rps4*, *rps7*(2), *rps8*
	Subunits of RNA polymerase	*rpoA*, *rpoB*, *rpoC1* ^a^, *rpoC*2
	Ribosomal RNAs	*rrn16*(2), *rrn23*(2), *rrn4.5*(2), *rrn5*(2)
	Transfer RNAs	*trnA-UGC* ^a^ (2), *trnC-GCA*, *trnD-GUC*, *trnE-UUC*, *trnF-GAA*, *trnG-UCC*, *trnG-UCC* ^a^, *trnH-GUG*, *trnI-CAU*(2), *trnI-GAU* ^a^ (2), *trnK-UUU* ^a^, *trnL-CAA*(2), *trnL-UAA* ^a^, *trnL-UAG*, *trnM-CAU*, *trnN-GUU*(2), *trnP-UGG*, *trnQ-UUG*, *trnR-ACG*(2), *trnR-UCU*, *trnS-GCU*, *trnS-GGA*, *trnS-UGA*, *trnT-GGU*, *trnT-UGU*, *trnV-GAC*(2), *trnV-UAC* ^a^, *trnW-CCA*, *trnY-GUA*, *trnfM-CAU*
Other genes	Maturase	*matK*
	Protease	*clpP* ^b^
	Envelope membrane protein	*cemA*
	Acetyl-CoA carboxylase	*accD*
	c-type cytochrome synthesis gene	*ccsA*
	Translation initiation factor	*infA*
unknown function	Conserved hypothetical chloroplast ORF	Ψ*ycf1*, Ψ*ycf15*(2)*, ycf1*, *ycf2*(2), *ycf3* ^b^, *ycf4*

^a^, gene containing a single intron. ^b^, gene containing two introns. (2), duplicated genes. Ψ, pseudogene.

**Table 3:** Voucher information and GenBank accession numbers for the 13 newly sequenced *Hydrocotyle* plastomes.

Taxon	Voucher Specimen	Locality	GenBank Accession
*H. calcicola*	XX Zhou 533 (GACP)	Puer, Yunnan, China	OM288648
*H. dielsiana*	SR Yi 626 (GACP)	Wushan, Chongqing, China	OM288647
*H. himalaica*	L Gu 007 (GACP)	Jiangkou, Guizhou, China	OM674716
*H. hookeri*	JX Yang 0428 (GACP)	Motuo, Tibet, China	OM674717
*H. hookeri* subsp. *chinensisis*	QT 048 (GACP)	Kunming, Yunnan, China	OM674718
*H. hookeri* subsp. *handelii*	L Gu 005 (GACP)	Panzhou, Guizhou, China	OM674719
*H. javanica*	GX Hu 746 (GACP)	Wangmo, GuiZhou, China	OM674725
*H. peltiformis*	GX Hu 637 (GACP)	Lushui, Yunnan, China	OM674720
*H. ranunculoides*	L Qu 35 (GACP)	Kunming, Yunnan, China	OM692359
*H. salwinica*	GX Hu 638 (GACP)	Lushui, Yunnan, China	OM674721
*H. sibthorpioides* var. *batrachium*	L Gu 004 (GACP)	Huaxi, Guizhou, China	OM674723
*H. sibthorpioides* ‘Variegata’	C Ye 423 (GACP)	Beijing, China (Cultivated)	OM674722
*H. wilfordii*	L Gu 006 (GACP)	Dushan, Guizhou, China	OM674724

## Data Availability

The *Hydrocotyle* plastomes generated in this study are available in the NCBI repository https://www.ncbi.nlm.nih.gov (accessed on 1 September 2023) with the accession numbers in Table 3.

## Supplementary Materials

The following supporting information can be downloaded at: https://www.mdpi.com/article/10.3390/ijms242316629/s1.
