# Supplementary figures and images for: New Insights into Phylogenetic Relationship of Hydrocotyle (Araliaceae) Based on Plastid Genomes
Source: Int J Mol Sci. 2023 Nov 22;24(23):16629. doi: 10.3390/ijms242316629 (PMC10706649; doi:10.3390/ijms242316629)

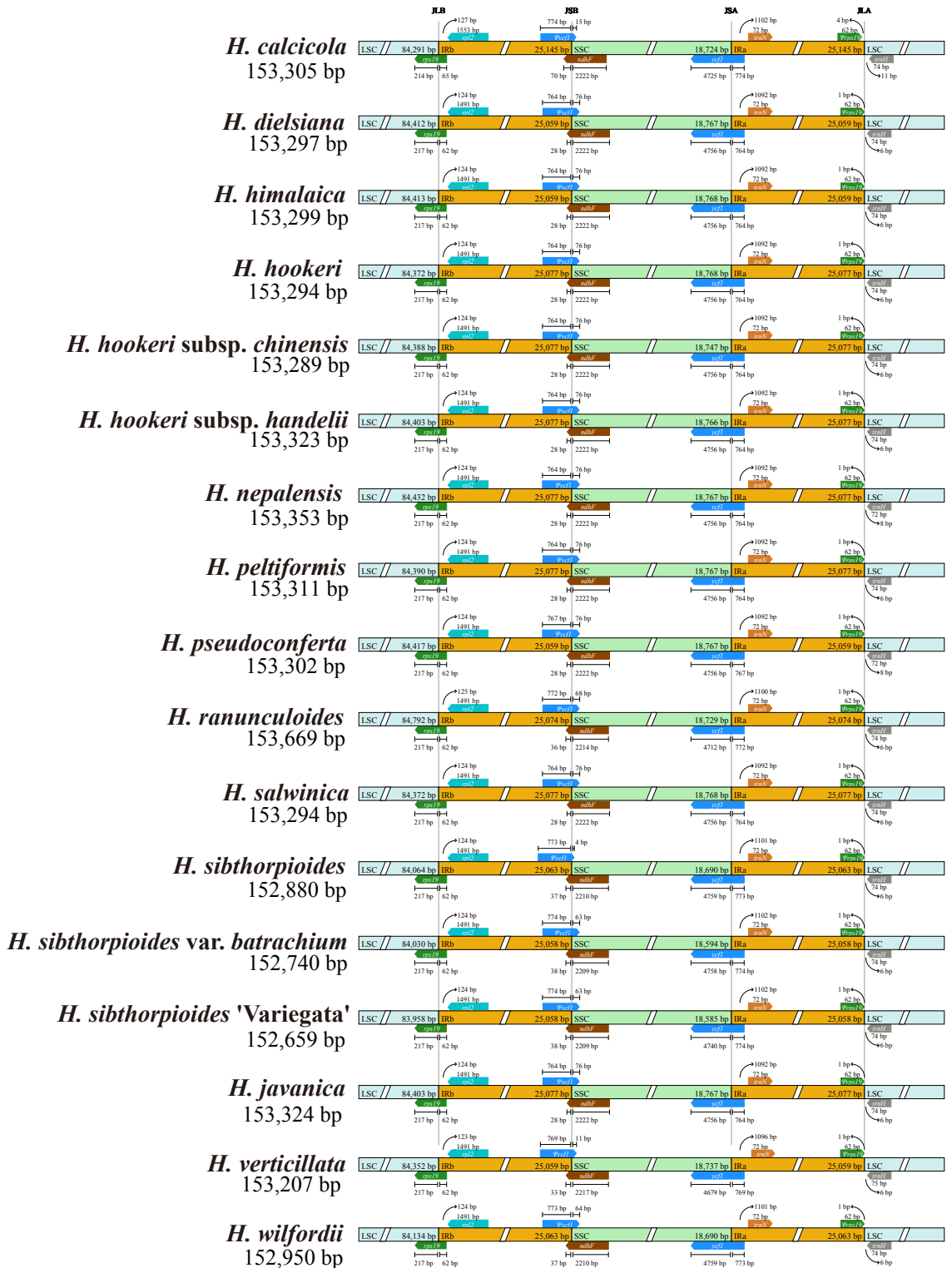

Supplement: Supplementary file 1 [file ijms-24-16629-s001.zip › Figure S1.pdf]

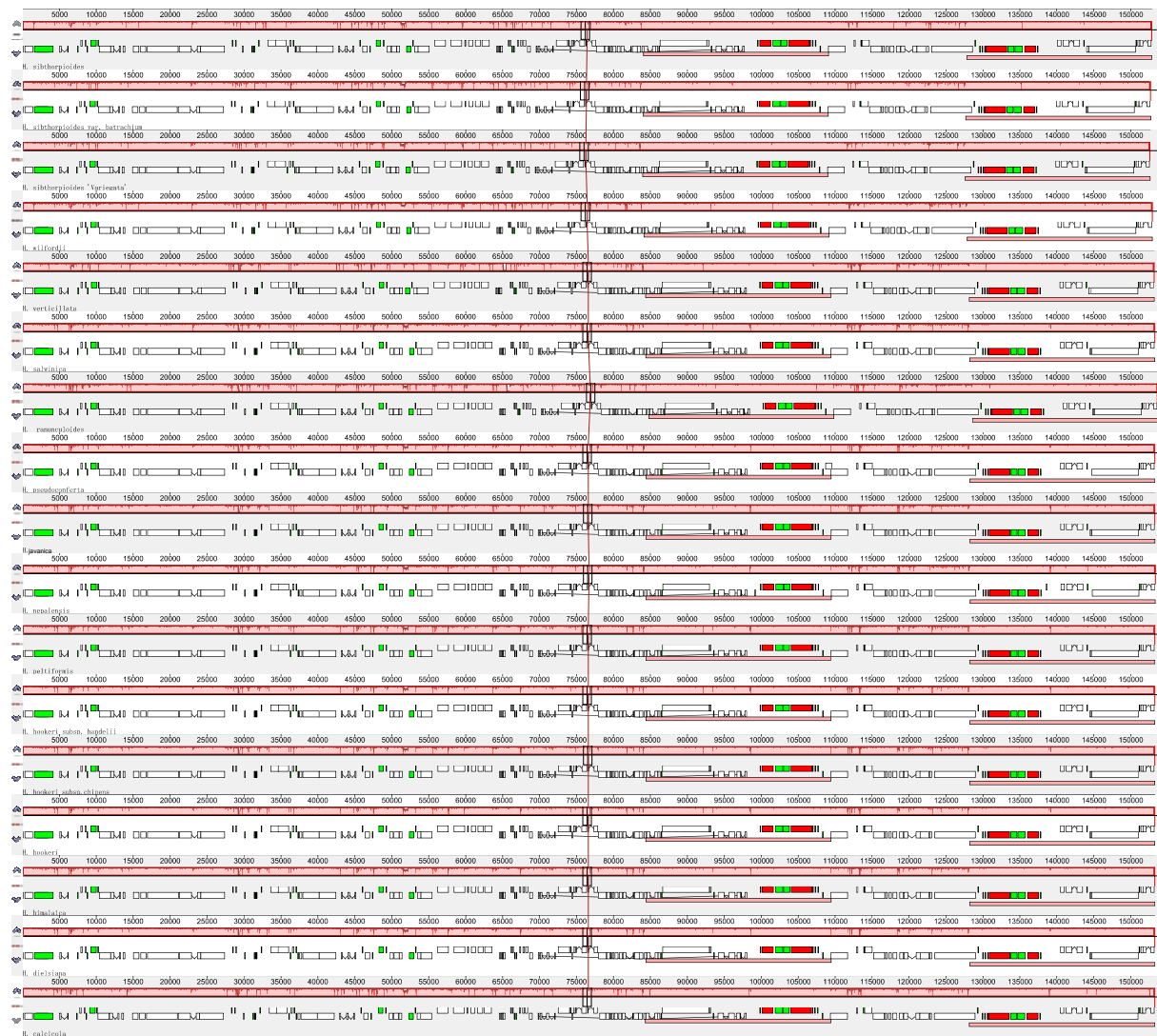

Supplement: Supplementary file 1 [file ijms-24-16629-s001.zip › Figure S2.pdf]

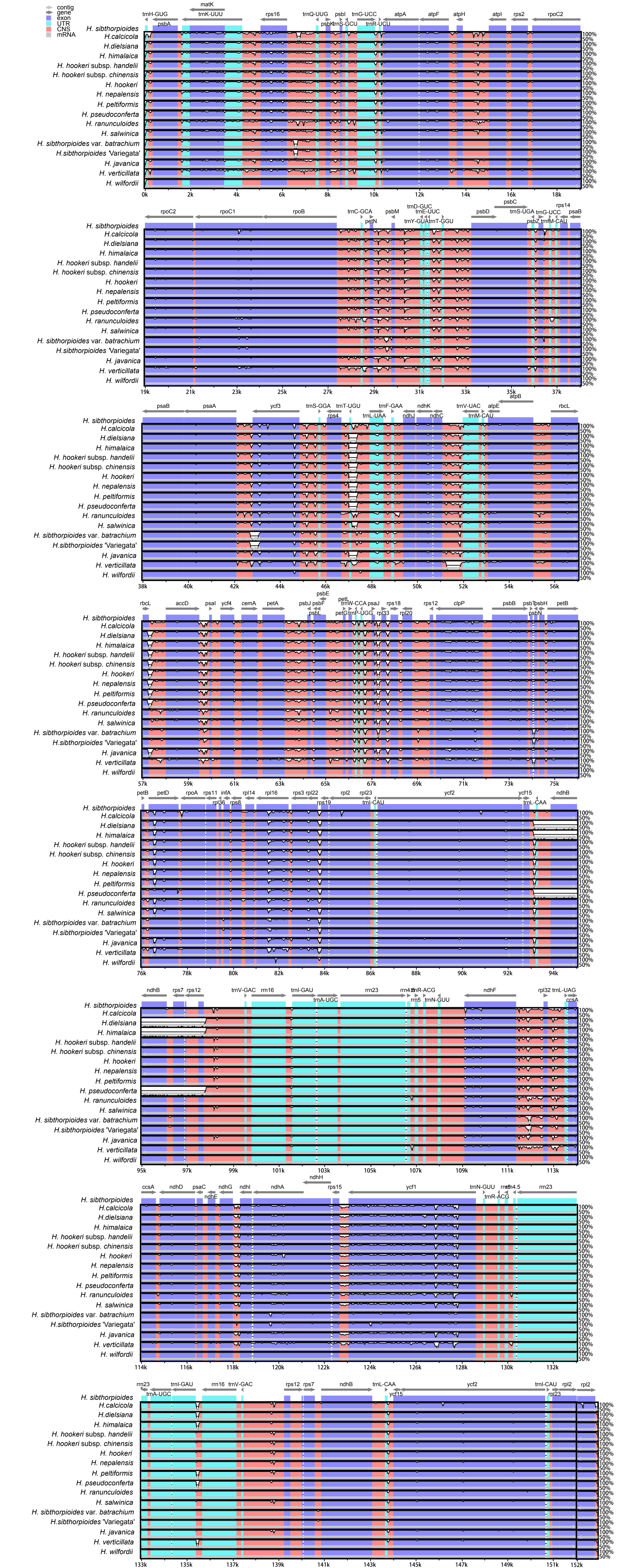

Supplement: Supplementary file 1 [file ijms-24-16629-s001.zip › Figure S3.pdf]
